# Exploring acceptability, opportunities, and challenges of community-based home pregnancy testing for early antenatal care initiation in rural Kenya

**DOI:** 10.1186/s12889-024-19254-7

**Published:** 2024-06-29

**Authors:** Lilian Otiso, Yussif Alhassan, Tom Odhong, Boniface Onyango, Nelly Muturi, Charlotte Hemingway, Lois Murray, Emily Ogwang, Linet Okoth, Mandela Oguche, Vicki Doyle, Nadia Fomuso, Miriam Taegtmeyer

**Affiliations:** 1 Department of International Public Health, Liverpool School of Tropical Medicine, Pembroke Place, Liverpool, L3 5QA UK; 2 LVCT Health, Nairobi, Kenya; 3 Migori County, Migori, Kenya; 4 Migory County, Migori, UK; 5 Airbel Impact Lab- International Rescue Committee, Nairobi, Kenya; 6 UK Health Security Agency, London, UK; 7 Department of Clinical Sciences, Liverpool School of Tropical Medicine, Liverpool, UK; 8 Tropical Infectious Diseases Unit, Liverpool University Hospitals Foundation Trust, Liverpool, UK; 9 LVCT Health, Homabay, Kenya

**Keywords:** Home pregnancy testing, Pregnancy self-testing, Community pregnancy testing, Community health workers, Early antenatal care, Rural setting, Kenya

## Abstract

**Background:**

Many women in low- and middle-income countries, including Kenya, access antenatal care (ANC) late in pregnancy. Home pregnancy testing can enable women to detect pregnancy early, but it is not widely available. Our study explored the acceptability and potential of home pregnancy testing delivered by community health volunteers (CHV) on antenatal care initiation in rural Kenya.

**Methods:**

This study was part of a public health intervention to improve uptake and quality of ANC. Between November and December 2020, we conducted 37 in-depth interviews involving women who tested positive or negative for a urine pregnancy test provided by CHVs; CHVs and their supervisors involved in the delivery of the pregnancy tests; facility healthcare workers; and key informants. Using Sekhon et al.‘s framework of acceptability, the interviews explored participants’ perceptions and experiences of home pregnancy testing, including acceptability, challenges, and perceived effects on early ANC uptake. Data were analysed thematically in NVivo12 software.

**Results:**

Home pregnancy testing was well-received by women who trusted test results and appreciated the convenience and autonomy it offered. Adolescents cherished the privacy, preferring home testing to facility testing which could be a stigmatising experience. Testing enabled earlier pregnancy recognition and linkage to ANC as well as reproductive decision-making for those with undesired pregnancies. Community delivery of the test enhanced the reputation and visibility of the CHVs as credible primary care providers. CHVs in turn were motivated and confident to deliver home pregnancy testing and did not find it as an unnecessary burden; instead, they perceived it as a complement to their work in providing ANC in the community. Challenges identified included test shortages, confidentiality and safeguarding risks, and difficulties accessing facility-based care post-referral. Newly identified pregnant adolescents hesitated to seek ANC due to stigma, fear of reprimand, unwanted parental notification, and perceived pressure from healthcare workers to keep the pregnancy.

**Conclusion:**

Home pregnancy testing by CHVs can improve early ANC initiation in resource-poor settings. Mitigating privacy, confidentiality, and safeguarding concerns is imperative. Additional support for women transitioning from pregnancy identification to ANC is essential to ensure appropriate care. Future research should focus on integrating home pregnancy testing into routine community health services.

## Background

Timely access to antenatal care (ANC) during pregnancy is vital for the health and wellbeing of both the mother and newborn [1]. The World Health Organization (WHO) recommends a minimum of eight contacts with a healthcare practitioner, with the first contact occurring within the first 12 weeks of gestation, to reduce perinatal mortality rates and improve women’s experience of care during pregnancy [2]. However, research conducted in various sub-Saharan African countries suggests a significant proportion of women delay their first antenatal care (ANC) visit, with many not seeking care until after the recommended first trimester [3]. The reasons for the delay in ANC initiation are multifactorial and associated with various personal, healthcare system, socio-economic, and cultural factors [3, 4]. A primary barrier to early ANC is the lack of access to pregnancy tests for early detection of pregnancy [5]. Early detection is crucial for women to access timely and appropriate antenatal care, which can help prevent adverse pregnancy outcomes [2].

In Kenya, access to appropriate and timely ANC remains a challenge for many women, especially those in rural areas. In 2020, only about 22% of women in Migori County in Western Kenya self-presented early enough in pregnancy (first 16 weeks of gestation) to fully benefit from testing and treatment of common conditions [6]. This delay in ANC attendance, combined with a shortage of testing commodities, often lead to delayed diagnosis of conditions such as HIV, syphilis, anaemia, and malaria, which contribute to adverse pregnancy outcomes. Kenya’s devolved healthcare system relies on community health volunteers (CHVs) to improve uptake of ANC services. These volunteers are selected from the community and identify and refer pregnant women to primary care facilities, offering counselling and other low risk maternal/reproductive health services [7]. CHVs lack the resources for routine pregnancy testing and are expected to identify pregnant women through conventional means, like self-reports of missed periods or pregnancy-related symptoms such as morning sickness, frequent urination, nausea, and vomiting.

There is growing evidence of the potential for pregnancy self-testing to facilitate early ANC attendance and improve maternal health [8–11]. The WHO Consolidated Guideline on Self-Care Interventions for Health recommends pregnancy self-tests can be a reliable and effective option for women to confirm pregnancy [12]. However, access and use of pregnancy self-tests have remained low and inequitable in resource-constrained settings, with tests not available or affordable for women in rural areas [9, 13]. Such barriers can constrain women’s autonomy over their reproductive plans and decisions. Alternative delivery approaches of pregnancy self-tests have been suggested that leverage community structures and are integrated into the formal health system. This approach has been successfully implemented in pilot projects in Uganda, Kenya and Madagascar among others, where community health workers (CHWs) have been trained to provide home-based services, including pregnancy self-tests [14, 15]. CHWs have been widely noted for their vital role in providing home-based services in many low-income countries [16], suggesting their potential to improve access to pregnancy testing in rural settings.

However, the acceptability of CHW delivery of home pregnancy testing in low-income settings, by communities, health workers and policy makers, remains unclear and contested [13, 17, 18]. Our study explored stakeholders’ perceptions and experiences of CHVs delivery of home pregnancy testing, including acceptability, challenges, and perceived effects on early ANC initiation. The findings will contribute to the body of knowledge on the feasibility of CHW delivery of home pregnancy testing and provide recommendations for improving community-based maternal and reproductive health services in low-income settings.

## Methods

The intervention was implemented between November 2019 and December 2020 but was interrupted by COVID-19 lockdown from March to June 2020 in the county.

### Study design

This study is part of a larger pilot project called *4byFour*, which aimed to improve uptake and quality of ANC in Migori County, western Kenya. The model name *4byFour* describes its target of 4 tests (syphilis, anaemia, malaria and HIV) by four months (of pregnancy) and 4 (ANC) visits for all women as per Kenyan national policy at the time of implementation [19]. The data presented in this paper is a subset of the larger study on the impact of the *4byFour* model on the utilisation and quality of ANC, findings of which as being reported elsewhere. A descriptive qualitative design was employed to better understand participant perspectives and the context in which the views were embedded [20]. Data collection was cross-sectional involving mainly in-depth interviews.

We employed Sekhon et al.‘s framework of acceptability to explore the study topic [21]. The framework defines acceptability as a multi-faceted construct that reflects the extent to which a healthcare intervention is considered appropriate by those delivering or receiving it. It consists of seven dimensions: affective attitude, burden, perceived effectiveness, ethicality, intervention coherence, opportunity costs, and self-efficacy. We deemed this framework to be well-suited for comprehensively exploring the dynamic nature of CHV delivery of home pregnancy testing. The framework was used to inform the design of the topic guides as well as to frame emerging themes from the data.

### Study settings

The study was conducted in Migori County, a mainly rural county in western Kenya, which has ten sub-counties and a population of about 1.1 million in 2019 [22]. Administratively, the county is divided into multiple community health units (CHUs), which serve as the primary health service delivery units for around 5000 residents. These CHUs are linked to healthcare facilities like sub-county hospitals, health centres, and dispensaries. They are staffed by CHAs and CHVs, with the CHVs typically responsible for 50–100 households [23, 24]. The County has a high rate of adolescent mothers and ranks fourth in Kenya in terms of HIV prevalence with a rate of 13% compared to a national rate of 4.9% in 2018 [25]. The local economy relies heavily on subsistence farming, fishing, and small-scale mining, with basic promotive and preventive services provided at household level by a network of over 2000 CHVs. The County was purposively selected for the study based on the high maternity morbidity and low proportion of women attending ANC in the first trimester of pregnancy(21%) [26, 27] ; well-established links with the County Health Management Team; and prior experience with community quality improvement approaches in some of its sub-counties. The intervention in two facilities focused in Arombe and Godkwer and their linked CHUs. These were selected due to their geographical accessibility, existing links with the LVCT-Health team, and presenting different sizes and patient flow.

### Intervention description

*4byFour* aimed to improve antenatal care by enhancing community referral in early pregnancy and enhancing the availability and quality of healthcare services in healthcare facilities. The project had three overlapping components: enhancing integrated point-of-care testing, building the capacity of community-facility work improvement teams to use data for quality improvement, and implementing community pregnancy testing and referral.

The community pregnancy testing component aimed to enhance the capacity of CHVs to identify, educate, refer, and encourage pregnant women to attend ANC early. This intervention targeted CHVs and their supervisors (Community Health Assistants, CHAs) working in the catchment locations of participating community units (CU) in Migori County. CHVs were trained in pregnancy mapping, distribution, and testing and interpretation of simple urine pregnancy tests, which were not readily available to the rural poor and were rarely seen outside health facilities in western Kenya [15]. The two-day training included lectures, group work, demonstrations, role plays, Q&A sessions. Training topics included components of the pregnancy test kit, urine sample collection, reading and interpretation of test results and how to safely dispose used pregnancy test kits. The training included how to approach women and adolescents who were eligible for the pregnancy test, how to support women and girls who tested positive to ensure the most appropriate measures for disclosure and referral to a health facility for ANC services and any additional support if required. Once trained, CHVs disseminated information about the availability of the tests and went with them during their regular healthcare visits to households. Each CHV received 10 pregnancy test kits and could replenish their stock if needed. These were replenished/restocked weekly to avoid any testing kits storage and handling interferences. Target beneficiaries included all women of reproductive age (15–49 years) within the CU. CHVs employed key questions/criteria to check if a woman/girl was eligible for the test, such as whether they had missed their period in the last month or had pregnancy-related symptoms like morning sickness, frequent urination, nausea, vomiting, etc. CHVs obtained verbal consent from women/girls before giving out tests. These tests were provided free of charge, and interested women were given a specimen bottle along with information on how to use and read interpret the results. The women would then administer the test and read the results at their convenience and receive any support required from the CHV after providing a urine sample. Women who tested positive were referred to the nearest health facility for ANC and for further support including counselling if required. A total of 78 CHVs and 6 CHAs participated in the training and were involved in the rollout of the pregnancy test in the community.

### Study participants and recruitment

A purposive sampling approach was employed to select study participants to provide rich and diverse data to effectively explore the research questions [20]. Participants were selected based on their experience and/or knowledge of community pregnancy testing and maternal health services in the county. This included pregnant and recent mothers, including adolescents (< 18 years), who had benefitted from the *4byFour* pregnancy test kits; CHVs and CHAs involved in delivering the pregnancy tests and referral, healthcare workers based at referral facilities, and County Health Management Team (CHMT) members. Several mothers were recruited as they visited healthcare facilities for ANC, while a few were identified through the community pregnancy mapping tool used by CHVs. CHVs and CHAs were recruited through their participation in the intervention. Male and female CHVs from different study sites were included to ensure representation. A total of 14 participants were included in the study (Table 1), with the sample size determined by data saturation [28].

**Table 1:** Overview of participant characteristics

Participant category	Female	Male	Total
Pregnant and recent mothers	14	0	14
Community health volunteers	6	2	8
Community health assistants	3	0	3
Facility health workers	1	2	3
Key informants (County Health Management Team members)	3	6	9
Total	**27**	**10**	**37**
Age (pregnant and recent mothers)			
< 18 years (adolescents)	7		
18 + years	7		

### Data collection

Data were collected through individual interviews to allow participants to speak openly and explore topics in greater depth [20]. Interviews were conducted in either English or Dhuluo (depending on the participant’s preference) between November and December 2020 by experienced qualitative research assistants (RAs) who had a deep understanding of the local culture and health system. RAs received further training in the study protocol and research ethics, including confidentiality and consent administration. Interviews were conducted face-to-face in safe and private locations within facilities and the community. Each interview lasted approximately 1 h and was audio recorded and complemented with written notes to ensure accuracy. Semi-structured topic guides were used to inform the interviews and explore a wide range of topics, such as participants’ perceptions and experiences of CHV delivery of maternal health services, including pregnancy testing, counselling, and referral. Additionally, the perceived effects of pregnancy home testing, factors influencing the uptake of pregnancy tests, as well as enablers and barriers to pregnancy mapping and CHV delivery of pregnancy tests, were included. Questions were piloted and iteratively revised as data collection evolved. Daily debriefings allowed for regular review and revision of topic guides.

### Data analysis

Interviews were transcribed using a denaturalised approach to ensure accurate and complete data capture [29]. For interviews conducted in Dhuluo, translation into English was done concurrently during transcription. Thematic framework analysis was used to analyse the data assisted by NVivo12 software. To start the analysis, a coding framework was developed based on a sample of the transcripts, which was then piloted and revised to ensure all relevant themes were captured. Once finalised, each transcript was systematically analysed to identify relevant codes, categories, and themes [30]. Sekhon et al.‘s acceptability framework was applied to develop and structure of the themes. To ensure rigor and trustworthiness, transcripts were independently coded, compared, and discussed among the authors in consortium meetings [31]. Feedback obtained was subsequently integrated into the analysis.

## Results

The key findings from the data are described in themes along with illustrative quotes. The themes align with five dimensions of Sekhon et al.‘s acceptability framework: affective attitude, perceived effectiveness, burden, ethicality, and self-efficacy.

### Affective attitude

This refers to the feelings and attitudes of both beneficiaries and CHVs towards the home pregnancy test.

#### Convenience, affordability, and empowered reproductive decision-making

The home pregnancy test was well-received by women of reproductive age in the community, including adolescents, as evidenced by CHV’s reporting high uptake of the test among women who were offered. Most women were motivated to undergo the test after their periods were late and they had experienced pregnancy symptoms, rather than to confirm a previous test. Women’s desire for the pregnancy test was largely related to its convenience and low cost, as noted by one woman in Arombe, “you sometimes suspect the pregnancy, but you delay going to the hospital because it is too far, you don’t have time, and the money. But if the CHV is around, you can do the test there and then, that is why I like it.” (Woman, 18 + years, Arombe). Several others mentioned the immediate test results reducing the “stress and anxiety” of a suspected pregnancy. A few adolescent girls mentioned the test providing privacy, avoiding the “embarrassment” of visiting a health centre where they might be scolded for being pregnant at an early age. Another benefit frequently reported by women was that it allowed them to make earlier reproductive decisions. One woman in GodKwer stated, “For us, when you know your results early, then you can make a decision whether you want to keep the pregnancy or get rid of it. You get pregnant this week; you do the test the following week…so you can start preparing what to do.” (Woman, 18 + years, GodKwer).

At the community level, there were no significant complaints about the home pregnancy testing, and the community was reported to be mostly supportive of its introduction. A CHV in Masara reported positive feedback from the community, stating, “They appreciate that we have done good with the pregnancy test… I haven’t seen them complain.” (CHV, Masara).

#### Women trusted home pregnancy tests and favoured CHV involvement

Before the intervention, most women in the community were unaware of home pregnancy tests and believed hospitals were the only place where they could be tested for pregnancy. However, they had confidence in the effectiveness of the tests and trusted the results. As one woman from Arombe noted, “I believe in it… Last time the CHV gave it to me, and when I tested, the lines showed. He told me to go to the health centre, and when they checked, they confirmed it.” (Woman, 18 + years, Arombe). Several mothers expressed willingness to use home pregnancy tests if they were provided through CHVs, as they had faith in them and had received their support over the years: “They [CHV] should give it to us so that we can get it when we need it. … they have been supporting us all these years. … I like them to be around because they [CHV] help those of us who have not been to school to read the results.*”* (Woman, 18 + years, GodKwer). CHVs noted performing the test on-site allowed for counselling and support to access the necessary care at the health centre. The majority of CHVs supported the integration of home pregnancy testing into their routine work, seeing it as an additional tool to enhance referral for early ANC.

### Self-efficacy

Self-efficacy pertains to the perceived ability and confidence of both beneficiary women and CHVs in using and interpreting pregnancy tests, as well as in providing necessary counselling and support for women.

#### Some women’s limited confidence and ability to self-administer the pregnancy tests

While several women demonstrated the correct technique for administering and interpreting the test, some adolescent girls preferred self-administration due to privacy concerns, while older women (18 + years) occasionally lacked confidence and preferred CHV assistance with using and interpreting the test. Although CHVs were expected to provide brief training to women on how to use the test, some women still felt unsure and wanted CHVs present for support. One woman stated, " sometimes you worry that you will make a mistake. If the CHVs are there to help, that will be good…it is new to us.” (Woman, 18 + years, Arombe). Some CHVs expressed concern that many women would not know how to use the test correctly and might rely on a false result. Both types of participants suggested women would need extensive train on how to use the kits and read the test results if they were introduced to home pregnancy tests.

#### CHVs limited counselling skills on undesired pregnancy

The CHVs reported competence and confidence in identifying potential pregnant women and conducting pregnancy tests. One CHV from Wiga reported, “It [pregnancy test] is not too hard, it is easy for all of us. The training they gave us was good. … I can do the test, teach them [women] how to use it, and refer those who are positive. … they also taught us how to identify somebody who is pregnant even when it is not showing”. (CHV, Wiga). However, CHVs lacked the knowledge and skills required to counsel women who test positive but do not desire their pregnancy. The CHVs expressed confusion and concern when they encountered such cases, and noted women often resorted to unsafe abortion practices as a result. As a CHV from Masara noted, “We get confused if the woman doesn’t want the pregnancy… sometimes you can test someone, and they start crying because they didn’t want to get pregnant” (CHV, Masara). CHVs requested more training to improve their counselling skills on how to support women with undesired pregnancy.

### Perceived effectiveness

Perceived effectiveness encompasses participants (CHVs, beneficiaries, HCWs and county stakeholders) perceptions of both the negative and positive impact of home pregnancy testing on maternal and reproductive care and health.

#### Improved pregnancy detection and earlier referral and ANC attendance

Both women and CHVs expressed a shared view that the introduction of home pregnancy tests contributed to the early detection of pregnancy within the community. Many women reported being able to confirm pregnancies earlier than before, enhancing their ability to access health education and ANC services. As an adolescent woman from Arombe stated, “It has brought ANC clinic closer to us in the community, so we are able to know our pregnancy status earlier.” (Adolescent mother, Arombe). CHVs noted the tests improved their ability to identify pregnant women earlier and effectively, without having to rely solely on physical attributes. As one CHV from Godkwer stated, “We have teenagers in the community, and especially with the coronavirus pandemic, some get pregnant, and will not admit they are pregnant because they know you will nudge them to go to the clinic and start ANC. But with the kits, they cannot lie to us, we would know, unlike before when you’d only know when the baby bump is big.” (CHV, Godkwer). The CHVs recognised the pregnancy test was “*the missing tool needed to perform our [their] work effectively*” (CHV, Arombe).

The early identification of pregnancy was noted to have improved ANC referral, particularly among adolescent girls who may initially deny being pregnant. Previously, CHVs could only refer women who were obviously pregnant, typically in the advanced stage of pregnancy. However, the introduction of home pregnancy tests enabled CHVs to confirm suspected pregnancies earlier, provide counselling, refer women to appropriate facilities, and encourage attendance at ANC. A CHV from Masara noted, “the other day a lady was laughing that this time I have insisted that she goes to the clinic early, yet she is just 4 weeks pregnant… She told me that for the first child she started at 6 months, so this time I have insisted on her going early.” (CHV, Masara). Participants noted the availability of tests attracted more women at risk of pregnancy to CHVs who leveraged the opportunity to provide ANC and family planning counselling and referral. CHVs reported women were more receptive to ANC counselling and took referral seriously if it was backed by a positive pregnancy test result, leading to improved referral attendance. As a CHV from Godkwer explained, “Previously you’d give women referrals, but they didn’t come but nowadays they do. More women find it easier to come to the clinic at an early stage after confirmation of the pregnancy because she gets convinced by the information, she finds that it is urgent.” (CHV, Godkwer).

Facility-based health workers confirmed an improvement in early ANC attendance following the rollout of the intervention. As one healthcare worker from GodKwer noted, “Currently there is a change…. In fact, they [ home pregnancy tests] have enhanced referral compared to before. Previously…there were very few cases of ANC mothers being referred by CHVs.… those days they were referring only the obvious mothers. That is a mother who is at 30 weeks pregnant. But of late we have seen a trend whereby they are referring early cases around 16 to 18 weeks.” (Healthcare worker, Godkwer).

#### Perceived empowerment and increased effectiveness among CHVs

Respondents observed the community-based delivery of the tests had a positive impact on the reputation and visibility of CHVs in the community. CHVs perceived they were now recognised as credible primary healthcare providers able to provide some level of care in the form of pregnancy testing instead of just information and referral, which was previously their only role. This led to increased motivation and appreciation among CHVs as more women sought their services for pregnancy testing, as reported by this CHV from Arombe, “now they have interests in CHVs, they respect us more. … people know I conduct pregnancy tests, so the young girls come to me to say that they have missed their periods the previous month and they want tests. They now believe CHVs as level-one health providers in the community… when this happens you get motivated.” (CHV, Arombe). The availability of the test led to increased interaction and engagement between CHVs and women, providing an opportunity for CHVs to educate women on various health issues. CHVs noted visiting women more frequently and were better able to track their progress throughout pregnancy and beyond. As one CHV shared, “the test has taken me out of sleeping at work like I used to. Because these people I am testing…. I have to keep reminding them and checking my book if so and so has gone back for the appointment. If she has not, I remind her. This thing has kept me busy.” (CHV, Masara).

### Ethicality

Ethicality pertains to ethical consequences of home pregnancy testing for both pregnant mothers and community based on individual and community values.

#### Privacy and safeguarding concerns

While many appreciate home pregnancy testing for its convenience and privacy, some women face privacy issues. This was especially true for adolescents who feared stigma and negative parental reactions. Lack of privacy sometimes resulted in women reporting false negative results, particularly when they must test in presence of their parents. As a result, many adolescents preferred to visit the CHV to obtain the test in a private location rather than have it delivered to their homes. As this woman noted, “at times the CHV will ask you to do the test when your parents are at home, so you have to find excuse, or you do it and tell him it is negative. … you fear that your parents will not be happy if they find that you are pregnant. … we like it but they should not give it us at home when our parents are around” (Adolescent mother, Masara). Both CHVs and women emphasised the need for additional training in confidentiality and safe testing management.

#### Concern about early pregnancy disclosure due to fears of supernatural harm/superstition

Many women were hesitant to share information about their pregnancy with CHVs, which made them reluctant to take up the pregnancy testing. This reluctance was often due to cultural norms that encourage delayed pregnancy disclosure. Women commonly preferred to keep their pregnancy secret, especially during the early stages, due to fear of potential supernatural harm. They believed if CHVs found out about their pregnancy, they might disclose the information to other people in the community, leading to unnecessary publicity around an unplanned pregnancy, which could potentially harm the baby or the mother’s reputation. As this woman reported, “I don’t always want people to know [about my pregnancy] when it is still early. You don’t know who wishes you well and who doesn’t … you might lose it. It is better to keep it secret. … even with the CHV. … I initially refused to do the test when they gave it to me.” (Woman, 18 + years, GodKwer). Some adolescent mothers expressed distress regarding the stigma linked to teenage pregnancy, leading them to hesitate in disclosing their pregnancy. Most CHVs acknowledged these privacy concerns and recognised gaining the trust of their clients was essential to providing accurate testing and counselling services in the community.

#### Gender-related challenges to CHV provision of home pregnancy test

Several male CHVs expressed difficulties in conducting pregnancy tests in the client’s home, particularly when the woman’s partner was absent or at work. Male CHVs reported feeling uncomfortable discussing urine, reproduction, and sex-related issues with women, especially in the absence of their partners. A male CHV from Godkwer expressed this concern: " it [CHV delivery of pregnancy tests] is good, but it creates a hard time for male CHVs. When you go to a household and find the husband isn’t around, being found alone with the opposite sex can cause issues due to people’s imaginations. It creates tension and suspicion. Maybe the husband didn’t know about 4byFour, but he finds you telling his wife to bring a urine sample, making it hard.” (CHV, Godkwer). When probed, several women reported a preference for a female CHV to administer the pregnancy test. Some CHVs recommended working in pairs and raising awareness in the community about CHVs administering pregnancy test to reduce risks and enhance trust in the community.

### Burden

Burden refers to challenges associated with CHV-delivery of the home pregnancy tests.

#### Frequent shortage of testing commodities

The effective rollout of home pregnancy testing by CHVs was hindered by supply challenges, including a shortage of specimen bottles and gloves, as reported by some CHVs. Although gloves, and to a certain extent bottles, were not necessary for women who were self-testing, many CHVs preferred to wear gloves for hygiene purposes. This was particularly important when some women were collecting urine samples for the CHVs to assist them in performing the test and interpreting the results. Some CHVs appreciated wearing gloves to maintain a professional and clinical appearance while carrying out their duties. Due to the scarcity of containers for collecting urine, CHVs often resorted to recycling the same containers multiple times, which could have potentially compromised the accuracy of the test results. Some CHVs expressed concern about this issue, as highlighted by this CHV in Arombe: “What didn’t go so well is when we were given these test kits, we lacked containers for collecting urine. The containers could be used two times or even ten, so I feel this issue affected it. Sometimes you tell them the container should be washed but they tell you it should not be washed that it is you who came with it.” (CHV, Arombe).

#### Barriers to care following a positive pregnancy test result

Participants in the study highlighted various challenges related to accessing ANC services among women who tested positive for pregnancy. Financial constraints and long distances to healthcare facilities were commonly identified as major barriers to accessing ANC services among women who were referred to care, as noted by this participant “… the CHV advised me and asked me to go and see the nurses at the health centre…. I could not go immediately; it was 2 months before I went.… it is too far from us, and I did not have money and time.” (Woman, 18 + years, Arombe). Healthcare facility staff reported shortages of testing and reagents, which often resulted in incomplete or delayed ANC for pregnant women: “we have seen an improvement [in early ANC uptake] since 4byfour, but sometimes they come, and you don’t have the resources to provide full service….” (Healthcare worker, Godkwer). Stigma surrounding pregnancy, particularly among adolescent girls, emerged as a significant challenge, with fear of reprimand or reporting by facility staff cited as a deterrent to seeking ANC services early. Some adolescents reported avoiding healthcare facilities earlier in pregnancy due to concerns of being persuaded to keep the pregnancy or forced parental notification, often leading to delayed or missed ANC clinic attendance after CHV referral. CHVs also mentioned challenges in tracking ANC referrals due to communication and information-sharing limitations between CHVs, pregnant women, and healthcare facilities.

## Discussion

This study found home pregnancy test delivered by CHVs is well-received by women in rural Kenya, including adolescents. It enabled early pregnancy detection, improved reproductive decision-making, and enhanced ANC linkage, potentially increasing early ANC attendance. The community delivery approach empowered CHVs, who perceived their credibility and visibility as primary care providers to have been enhanced. While CHVs showed willingness to integrate home pregnancy testing into their routine work, challenges such as privacy concerns, test shortages, and follow-up care issues remain. The issue of supporting women who don’t want to continue a pregnancy in a context where abortion is illegal remains a problem. Addressing such challenges is essential to maximising the benefits of home pregnancy testing in low-income settings.

Women’s positive response to the home pregnancy testing indicates a preference for this self-care approach to maternal and reproductive healthcare. This finding corroborates previous studies showing women in low-income settings prefer self-testing to clinic-based testing for convenience, privacy, and empowerment even though both are available free of charge [13, 14, 32]. Pregnancy self-testing increases women’s autonomy and agency over their reproductive choices, including whether to keep the pregnancy or not [8, 33], as was demonstrated in our study. Home pregnancy testing addresses some of the barriers to women accessing testing services, such as distance, cost, and stigma [34]. However, women’s autonomy and agency over their reproductive choices may be constrained by limited access to abortion services. Feedback from women in this study suggests those with an undesired pregnancy may desire a termination, despite the illegality of abortion in Kenya [35]. CHVs concerns about their inability to support women with undesired pregnancies after a positive test result indicates the need to empower them with skills and training to assist women, focusing on empathy, confidentiality, and avoiding imposing their values [14].

The perceived impact of home pregnancy testing on ANC uptake aligns with prior research [8, 33] and indicates its potential to reduce delayed ANC initiation [8, 14]. In our study, improved ANC uptake was facilitated by early pregnancy detection and positive test results, particularly among women at risk of late detection, as well as enhanced counselling, referral, and follow-up by CHVs. However, women who test positive still faced barriers to accessing care, including transportation, financial constraints, stigma, sociocultural factors, limited knowledge of the correct timing and benefits of early and frequent ANC attendance, and inadequate services in facilities. Continuous education, counselling, and support for women as well as training of CHVs are essential to optimising the benefits of CHV delivery of home pregnancy testing. Additional support is necessary to enhance the quality of care in linked facilities, along with community campaigns to address pregnancy-related stigma and inimical sociocultural practices [36]. Further, commodity shortages for pregnancy testing, including gloves, containers, and safe disposal, must be addressed when scaling up CHV-delivered home pregnancy testing [14].

The study highlights challenges and opportunities of home pregnancy testing models: self-testing and CHV-assisted testing. Self-testing appealed more to adolescents than other women (18 + years old) due to privacy, while women (mainly 18 + years), seemed to prefer CHV-assisted testing. The involvement of CHVs in interpreting test results presented an opportunity to encourage women who test positive to seek ANC but raised privacy and coercion concerns. This aligns with prior studies where older, less educated women showed less interest in self-testing technology due to literacy limitations [37]. Women (mainly 18 + years) may have distinct preferences such as privacy, confidentiality, autonomy, and empowerment. Extensive education and CHV support can address barriers to home pregnancy test use such as stigma, fear, and low confidence, especially among older, less literate women [38]. Consistent with Steege et al. [39], male workers administering tests posed acceptability and safeguarding challenges, as women preferred female CHVs. Male CHVs raised concerns about potential accusations of inappropriate relationships with female clients, emphasising the need for safeguarding measures prioritising clients’ and CHVs’ well-being in community health service delivery [39, 40]. Strategies such as using ID badges, pairing male CHVs with female CHVs, and increasing community awareness about the pregnancy testing initiative may reduce suspicion and enhance the confidence of male CHVs in administering pregnancy tests in the community.

Ethical concerns, following Sekhon et al.‘s framework, significantly impacted the uptake of the home pregnancy test. Privacy issues arise, particularly in rural, low-income settings, where cultural norms and superstitious beliefs discourage early disclosure of pregnancy [36, 41]. CHVs, though trusted, may not ensure confidentiality due to shared community ties [42]. To address these concerns, CHVs should receive extensive training on privacy and confidentiality. Standard operating procedures for ethical practice and confidentiality should be developed. Empowering women to initiate contact with CHVs or ensuring sensitive testing approaches by CHVs is essential. Training CHVs on teenage pregnancy challenges and ensuring informed consent is vital. Education on test administration and interpretation, secure access facilitated by CHVs, and electronic systems with SMS reminders can enhance privacy and acceptability [13, 14, 32].

The acceptability of CHV delivery of the pregnancy test in this study indicates that they could contribute significantly to ensuring women achieve the recommended eight ANC contacts with healthcare professionals during pregnancy, as per the National Guidelines for Quality Obstetrics and Perinatal Care in Kenya [43]. Despite successful implementation of home pregnancy testing in research programs, integration into routine health systems in Kenya has been slow. Cost and logistical challenges may pose as potential bottlenecks to integration [44], but evidence in this study suggests that CHVs can effectively deliver home pregnancy tests, thus enhancing accessibility and potentially reducing costs. Further research is required to examine different community delivery models, barriers to integration, and potential solutions to scale-up. Cost-effectiveness assessments of home pregnancy testing and different delivery models would provide valuable evidence to guide policy decisions regarding scaling up [13].

### Limitations

The cross-sectional design of the study only offered a snapshot of participants’ experiences of the intervention and home pregnancy testing; it limits our ability to make causal inferences. The study’s small sample size limits the generalisability of the findings beyond the study community. A longitudinal study and a randomised controlled trial could provide valuable insights into the potential impact of the intervention, particularly in terms of promoting early ANC attendance during pregnancy and reducing the gestational weeks at presentation. The study did not assess the cost-effectiveness of the home pregnancy test, access beyond the project period, or women’s willingness to purchase it. These factors could have provided valuable information for scaling the initiative and should be addressed in future studies. Participants’ perspective of the home pregnancy testing may have been influenced by their previous experience with other projects in the study setting that have utilised pregnancy self-testing in their programs. We did not interview male participants or other community leaders who might have provided community perspective on the intervention, given their influential role at the household. Although the purposive sampling approach and the small sample size provided an in-depth exploration, they may limit the generalisation of the results to a broader context. it is imperative to understand the equity dimensions of CHV delivery of the pregnancy test, including exploring the differential access and utilisation of the test results on women.

## Conclusion

This study demonstrates that home pregnancy testing, delivered by CHVs, is well-received by women in a rural Kenyan setting. Women prefer self-testing for privacy, convenience, and empowerment and the test facilitates early detection of pregnancy and referral for ANC for women at risk of delaying ANC initiation. The integration of home pregnancy testing into routine health systems in Kenya is essential. CHVs’ delivery of the test empowers them as credible community health care providers and provides an opportunity to link pregnant women to care and provide follow-up support. However, barriers such as financial constraints, transportation issues, social stigma, and sociocultural factors continue to present challenges in accessing care after a positive home pregnancy test. It is important to address these alongside privacy and confidentiality concerns to maximise the potential benefits of community delivery of home pregnancy testing and ensure their scalability and long-term sustainability. Further research is needed to explore different community delivery models, integration barriers, and cost-effectiveness assessments of home pregnancy testing.

## Data Availability

Data are available from the corresponding author on request.

## Abbreviations

ANC: Antenatal care
CHAs: Community health assistants
CHVs: Community health volunteers
CHWs: Community health workers
CUs: Community units
HCW: Healthcare workers
HIV: Human immunodeficiency virus
RAs: Research assistants
WHO: World health organization
